# Arterial Pressure Variation as a Biomarker of Preload Dependency in Spontaneously Breathing Subjects – A Proof of Principle

**DOI:** 10.1371/journal.pone.0137364

**Published:** 2015-09-03

**Authors:** Anne-Sophie G. T. Bronzwaer, Dagmar M. Ouweneel, Wim J. Stok, Berend E. Westerhof, Johannes J. van Lieshout

**Affiliations:** 1 Department of Internal Medicine, Academic Medical Center, Amsterdam, the Netherlands; 2 AMC Heart Center, Academic Medical Center, Amsterdam, the Netherlands; 3 Department of Anatomy, Embryology and Physiology, Academic Medical Center, Amsterdam, the Netherlands; 4 Laboratory for Clinical Cardiovascular Physiology, Center for Heart Failure Research, Academic Medical Center, Amsterdam, the Netherlands; 5 Edwards Lifesciences BMEYE, Amsterdam, the Netherlands; 6 MRC/Arthritis Research UK Centre for Musculoskeletal Ageing Research, Queen's Medical Centre, School of Life Sciences, University of Nottingham Medical School, Nottingham, United Kingdom; Hospital Sirio-Libanes, BRAZIL

## Abstract

**Objective:**

Pulse (PPV) and systolic pressure variation (SPV) quantify variations in arterial pressure related to heart-lung interactions and have been introduced as biomarkers of preload dependency to guide fluid treatment in mechanically ventilated patients. However, respiratory intra-thoracic pressure changes during spontaneous breathing are considered too small to affect preload and stroke volume sufficiently for the detection by PPV and/or SPV. This study addressed the effects of paced breathing and/or an external respiratory resistance on PPV and SPV in detecting preload dependency in spontaneously breathing subjects.

**Methods:**

In 10 healthy subjects, hemodynamic and respiratory parameters were evaluated during progressive central hypovolemia (head-up tilt). Breathing conditions were varied by manipulating breathing frequency and respiratory resistance. Subjects responding with a reduction in stroke volume index ≥15% were classified as having developed preload dependency. The ability for PPV and SPV to predict preload dependency was expressed by the area under the ROC curve (AUC).

**Results:**

A breathing frequency at 6/min increased the PPV (16±5% vs. 10±3%, p<0.001) and SPV (9±3% vs. 5±2%, p<0.001) which was further enhanced by an expiratory resistance (PPV: 19±3%, p = 0.025 and SPV: 10±2%, p = 0.047). These respiratory modifications, compared to free breathing, enhanced the predictive value of PPV with higher accuracy (AUC: 0.92 vs. 0.46).

**Conclusion:**

Under conditions of progressive central hypovolemia, the application of an external respiratory resistance at a breathing frequency of 6/min enhanced PPV and SPV and is worth further study for detection of preload dependency from arterial pressure variations in non-ventilated subjects.

## Introduction

In anesthesiology and intensive care medicine, fluid administration is the cornerstone of treatment for hypovolemia but the detection of a clinically relevant blood volume deficit remains difficult [1, 2]. Only 40–70% of critically ill patients respond to fluid administration with a clinically significant increase in stroke volume (SV) and cardiac output (CO) [3]. The substantial number of patients not responding to fluid therapy calls for physiological markers capable to predict preload dependency or fluid responsiveness—that is, responding to fluid administration by increasing SV (and CO) [4, 5]. Assessment of preload dependency would enable identification of those patients who would benefit from volume expansion whereas avoiding fluid overload [5, 6]. To this end, variations in arterial pressure, like systolic (SPV) and pulse pressure variation (PPV) have been proposed as biomarkers of central hypovolemia and with that preload dependency [3, 5, 7, 8]. These dynamic indices are based on respiration-induced changes in venous return [9, 10] and associated variations in left ventricular preload transferred to arterial pressure. Although it has been demonstrated in previous studies that these indices are of clinical value, their application remains limited to patients who are mechanically ventilated with high tidal volumes [7, 11–13]. In contrast, in spontaneously breathing critically ill patients, both PPV and SPV were not accurate in predicting fluid responsiveness by insufficient sensitivity (63% and 47%, respectively) [14]. Similarly, in mechanically ventilated patients with spontaneous breathing movements, PPV does not identify responders to fluid administration [15].

Mechanical ventilation induces considerable cyclic changes in intra-thoracic pressure affecting left ventricular preload. In contrast, during spontaneous breathing the magnitude of respiratory induced preload alterations is considered as being too small and variable between consecutive breaths [16, 17] and therefore unable to detect variations in blood pressure and left ventricular SV [14, 18].

This study tested the hypothesis that in spontaneously breathing subjects, paced breathing at a pre-set frequency with augmented breathing resistance enhances the magnitude and predictive value of PPV and SPV. We therefore set out to quantify the separate and combined effects of paced breathing and an external respiratory resistance on arterial pressure variations in detecting preload dependency.

## Methods

### Subjects

Eleven healthy volunteers (4 male), age 25 (22–30) years, height 176 (163–183) cm and weight 68 (52–72) kg, with no history of fainting and/or cardiac arrhythmia and not taking cardiovascular medication participated in this study. The subjects abstained from heavy physical exercise and caffeinated beverages 4 hours prior to the experiment. The study was approved by the Medical Ethics Committee from the Academic Medical Center (Amsterdam, the Netherlands) and written informed consent was obtained prior to the experiment.

### Experimental protocol

**Fig 1:**
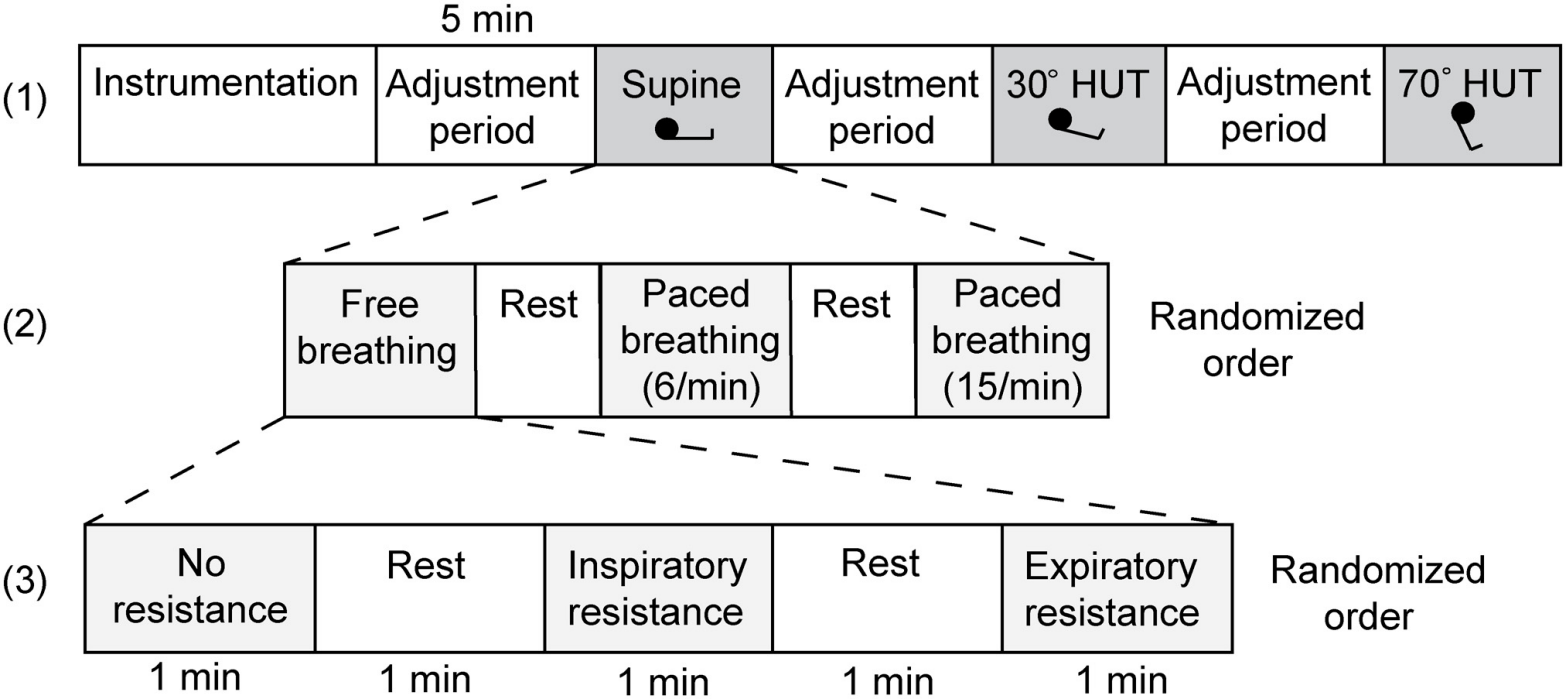
Experimental protocol. After instrumentation the measurements started with the subject in the supine position, followed by 30 and 70° head-up tilt with a 5 minute adjustment period in between (layer 1). Each test run encompasses three breathing conditions (layer 2, here only shown for the supine position) with and without an respiratory resistance (layer 3, here only shown for free breathing). The order of the breathing frequency and use of a respiratory resistance was randomized.

Measurements were performed with subjects positioned on a custom built computer controlled tilt table which minimizes confounding muscle tensing and vestibular stimulation [19]. In healthy humans the horizontal position provides for an 'optimal' central blood volume [20, 21] and therefore resting supine measurements represent normovolemic conditions whereas 30° (gravitational load 0.5 G) and 70° (0.9 G) passive head-up tilt (HUT) induces central hypovolemia [22, 23]. Following stepwise elevation of body angle, that body position was maintained for 5 minutes at respectively 30° and 70° to allow for hemodynamic adaptation. For each body position at the end of the stabilization periods, the volunteers performed nine frequency and resistance paradigms of breathing in a randomized order (Fig 1): free breathing, metronome paced breathing at 6/min and at 15/min, each without an external respiratory resistor, with an inspiratory resistor and with an expiratory resistor (7.5 cmH_2_O threshold resistor; ResqGARD, Advanced Circulatory Systems, Inc., Eden Prairie, MN). The 1 minute breathing periods were alternated with 1 minute of free breathing.

### Measurements

Blood pressure (BP) was continuously measured using a non-invasive volume clamp method (Nexfin, Edwards Lifesciences BMEYE, Amsterdam, the Netherlands). Left ventricular SV was estimated by a pulse contour method (Nexfin CO-trek, Edwards Lifesciences BMEYE, Amsterdam, the Netherlands) [24, 25] and CO was SV times heart rate (HR). SV index (SVI) was the ratio of SV and body surface area [26]. SPV and PPV were calculated from the BP signal:

$$100\times \frac{{\text{A}}_{\text{max}}-{\text{A}}_{\text{min}}}{({\text{A}}_{\text{max}}+{\text{A}}_{\text{min}})/2}$$ (1)

with A_max/min_ equal to, respectively, systolic arterial pressure (SAP) and pulse pressure (PP; SAP minus diastolic arterial pressure (DAP)). PPV and SPV were calculated for each breath and averaged over 5 consecutive breaths.

Airway flow and pressure were measured using an Alveotest flowmeter (Jaeger, Würzburg, Germany), tidal volume (TV) was the integral of airway flow (expressed in mL per kg predicted body weight) and end-tidal CO_2_ (PetCO_2_) was measured by capnography (Tonocap, Datex-Ohmeda, Madison, USA). Signals were visually inspected for artefacts and 60-second intervals were used for offline analysis (Matlab R2007b, Mathworks Inc. MA, USA).

### Statistical analysis

Data were analysed (Sigmaplot 11.0, Systat Software Inc., USA) and presented as mean ± SD. The hemodynamic effects of HUT and different breathing conditions were assessed using One Way Repeated Measures ANOVA with pairwise multiple comparisons (Holm-Sidak). To detect a relative difference ≥20% in PPV and SPV in response to changing breathing conditions, a sample size of 10 subjects was required (power 0.9; probability of type I error 0.05; two-sided significance level).

Subjects were assigned to two groups according to the percentage of the reduction in SVI following 30° HUT with reference to the supine value. A 15% increase in response to fluid administration is considered clinically relevant according to previously published data [14, 15]. In this study, subjects with a postural reduction in SVI≥15% following 30° HUT were classified as preload dependent. The ability for PPV and SPV to predict preload dependency was expressed as the area under the ROC curve (AUC). The AUCs were computed using the Hanley and McNeil method [27] and compared using a Z-test. A p-value ≤ 0.05 was considered to indicate a statistically significant difference.

## Results

All subjects completed the protocol. The data from 1 subject was excluded from analysis due to cardiac arrhythmia. As a result 10 subjects entered final analysis. With 30° HUT, 4 out of 10 subjects became preload dependent (ΔSVI≥15%). Increasing the gravitational load to 70° HUT, 4 other subjects become preload dependent while 2 subjects were still preload independent (ΔSVI < 15%).

### Hemodynamic and respiratory response to central hypovolemia

**Fig 2:**
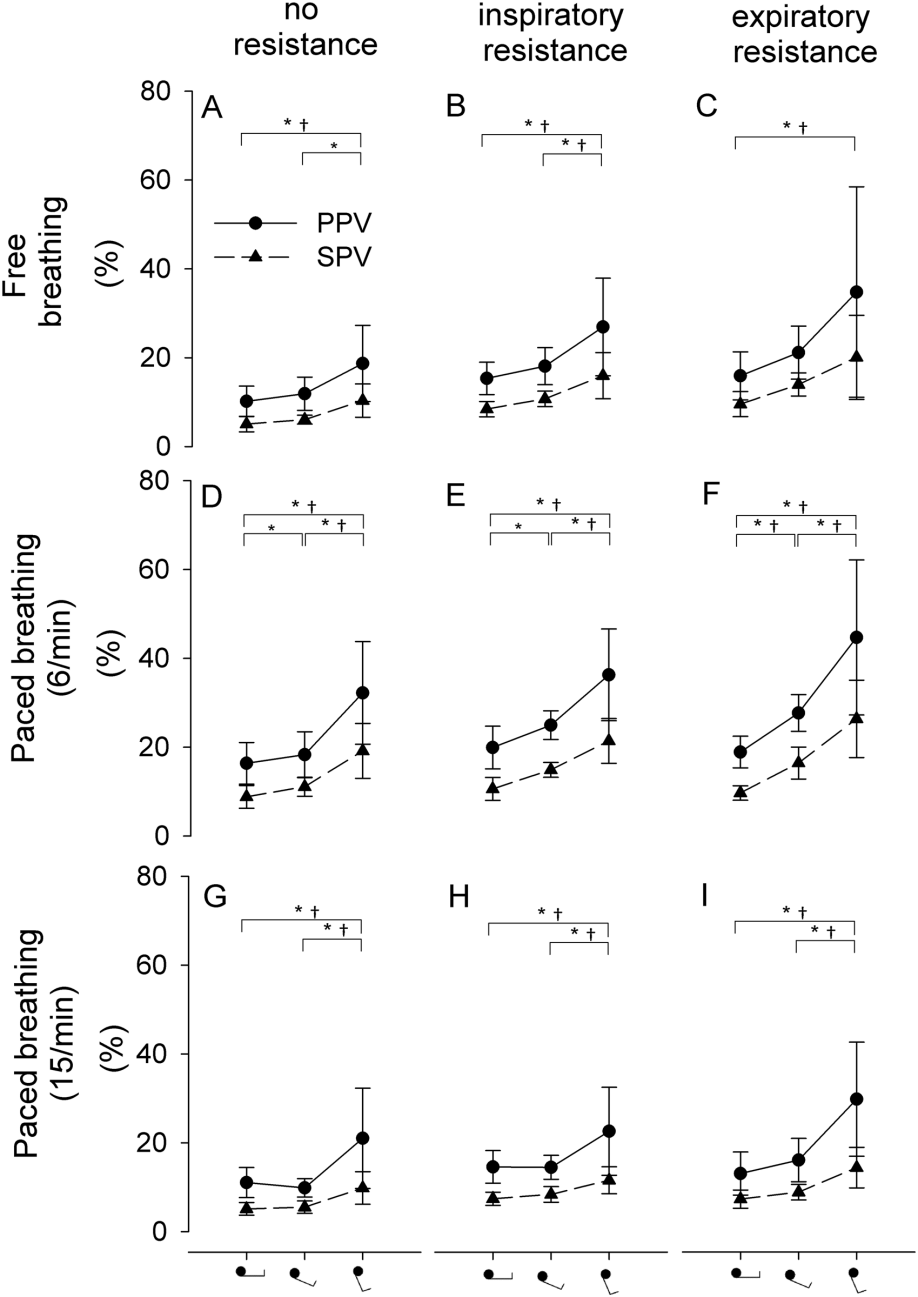
The influence of breathing frequency and respiratory resistance on pulse (PPV) and systolic pressure variation (SPV) during head-up tilt. Black dot/triangle: mean value ± SD. *p<0.05 for SPV; †p<0.05 for PPV.

The hemodynamic and respiratory response to graded central hypovolemia during free breathing without an external respiratory resistance is given in Table 1. PPV and SPV did not change from the supine position to 30° HUT and only SPV increased from 30 to 70° HUT (95±102%, p = 0.002; Table 2 and Fig 2A).

**Table 1:** Hemodynamic and respiratory response to head-up tilt.

		Supine	30° HUT	70° HUT
SAP	(mmHg)	120 ± 9	118 ± 8	118± 6
DAP	(mmHg)	71± 7	72 ± 6	78± 5
MAP	(mmHg)	88 ± 7	89 ± 7	93 ± 5
HR	(beats/min)	60 ± 11	60 ± 10	78 ± 13*
SV	(mL)	103 ± 13*	92 ± 11*	75 ± 10*
CO	(L/min)	6.2 ± 1.5	5.6 ± 1.3*	5.8 ± 1.2
TPR	(dyn.sec/cm^5^)	1206 ± 32	1358 ± 398*	1348 ± 346
AWP	(kPa)	0.33 ± 0.1	0.29 ± 0.15	0.41 ± 0.23*
ETCO_2_	(mmHg)	37 ± 4	36 ± 6	32 ± 5*
TV	(mL/kg)	12 ± 4	11 ± 2	13 ± 4

Average response during free breathing without an external respiratory resistance. AP, arterial pressure (systolic, diastolic and mean); HR, heart rate; SV, stroke volume; CO, cardiac output; TPR, total peripheral resistance; AWP, airway pressure; ETCO_2_, end-tidal CO_2_; TV, tidal volume.

*p<0.05 compared to the previous body position.

**Table 2:** Pulse and systolic pressure variation (PPV and SPV) for nine different breathing conditions during the supine position and 30° and 70° head-up tilt.

		No resistance			Inspiratory resistance			Expiratory resistance		
		Sup	30°	70°	Sup	30°	70°	Sup	30°	70°
Free breathing	PPV (%)	10±3	12±4	19±9	15±4†	17±4	26±11*	15±5†	20±6	33±24
	SPV	5±2	6±1	10±4*	8±2†	10±2	15±5*	8±3†	13±3	19±9
6/min PB	PPV	16±5†	18±5	32±12*	20±5†	25±3	37±10*	19±3†	28±4*	45±17*
	SPV	9±3†	11±2*	19±6*	11±3†	15±2*	22±5*	10±2†	17±4*	27±9*
15/min PB	PPV	11±3	10±2	21±11*	15±4†	14±3	23±10*	13±5	16±5	30±13*
	SPV	5±1	6±1	10±4*	7±1†	8±2	12±3*	7±2†	9±2	14±5*

PB, paced breathing.

*p<0.05 compared to the previous body position.

^†^p<0.05 compared to free breathing without a resistance in the supine position.

### Breathing frequency

No change in hemodynamic parameters or airway pressure was observed during paced (6 or 15 breaths/min) vs. free breathing (11±3 breaths/min) when subjects were in the supine position. A paced breathing frequency of 6/min but not 15/min increased tidal volume (20±5 vs. 12±4 mL/kg, p<0.001), PPV (16±5 vs.10±3%, p<0.05) and SPV (9±3 vs. 5±2%, p<0.05; Table 2)

In response to central hypovolemia, 6/min paced breathing increased SPV from supine to 30° HUT (9±3 vs. 11±2% p<0.05). From 30 to 70° HUT, PPV and SPV increased during both 6/min and 15/min paced breathing (PPV: 32±12 vs. 18±5% (p<0.001) and 21±11 vs. 10±2% (p = 0.001); SPV: 19±6 vs. 11±2% (p<0.05) and 10±4 vs. 6±1% (p<0.001), respectively), see Fig 2D and 2G.

### Respiratory resistance

In the supine position, adding either an inspiratory or expiratory respiratory resistance did not affect hemodynamic parameters or tidal volume whereas an increase was demonstrated in airway pressure (0.96±0.18 and 0.98±0.23 kPa vs. 0.33±0.16 kPa (p<0.001), respectively) and PPV / SPV (15±4 and 15±5 vs.10±3% (p<0.001) / 8±2 and 8±3 vs. 5±2% (p<0.001), respectively; Table 2). The application of either an inspiratory or expiratory resistance did not change PPV and SPV from supine to 30° HUT and only for an inspiratory resistance from 30 to 70° (PPV: 26±11 vs. 17±4%, p = 0.005 and SPV: 15±5 vs. 10±2%, p = 0.001), see Fig 2B and 2C.

### Breathing frequency & respiratory resistance

**Fig 3:**
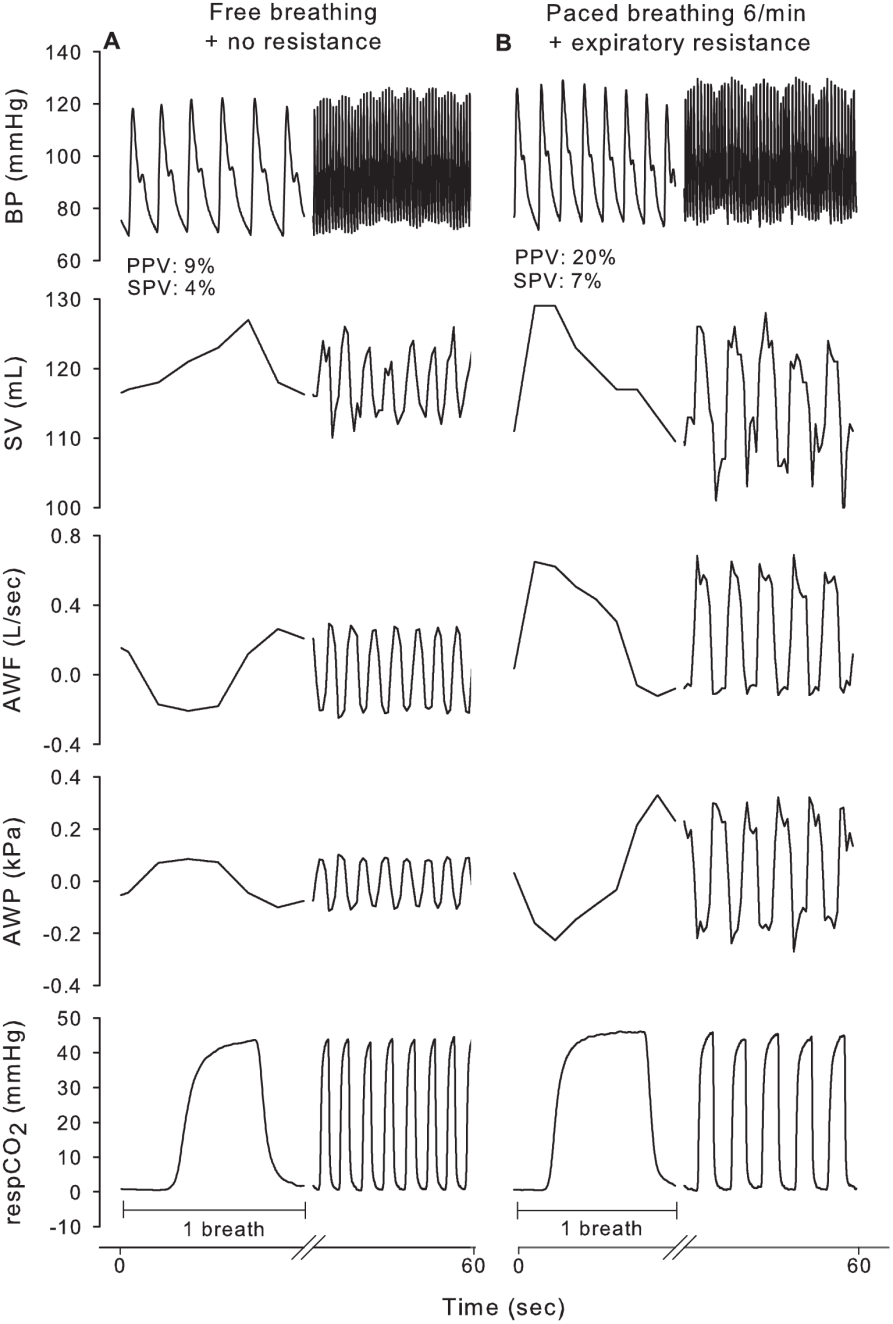
Illustration of the effect of 6/min paced breathing against an expiratory resistance for a single recumbent subject. AWF, airway flow; AWP, airway pressure; respCO_2_, respiratory CO_2_ partial pressure.

In the supine position, a combination of paced breathing at 6/min together with either an inspiratory or expiratory resistance further enlarged PPV (20±3 and 19±3 vs. 10±3% (p<0.001), respectively) and SPV (11±3 and 10±2 vs. 5±2% (p<0.001), respectively) compared to free breathing without an external respiratory resistance (Table 2 and Fig 3). In response to graded central hypovolemia, a 6/min paced breathing frequency together with an expiratory resistance increased PPV and SPV from 0 to 30° and from 30 to 70° HUT (19±3 to 28±4% (p = 0.05) to 45±17% (p<0.001) and 10±2 to 17±4% (p = 0.006) to 27±9% (p<0.001), respectively), see Fig 2F.

Adding an inspiratory resistance to a 15/min paced breathing frequency only increased PPV (15±4 vs. 10±3%, p = 0.005) in the supine position with no further discrimination in response to HUT.

### Predictive value of arterial pressure variations

**Fig 4:**
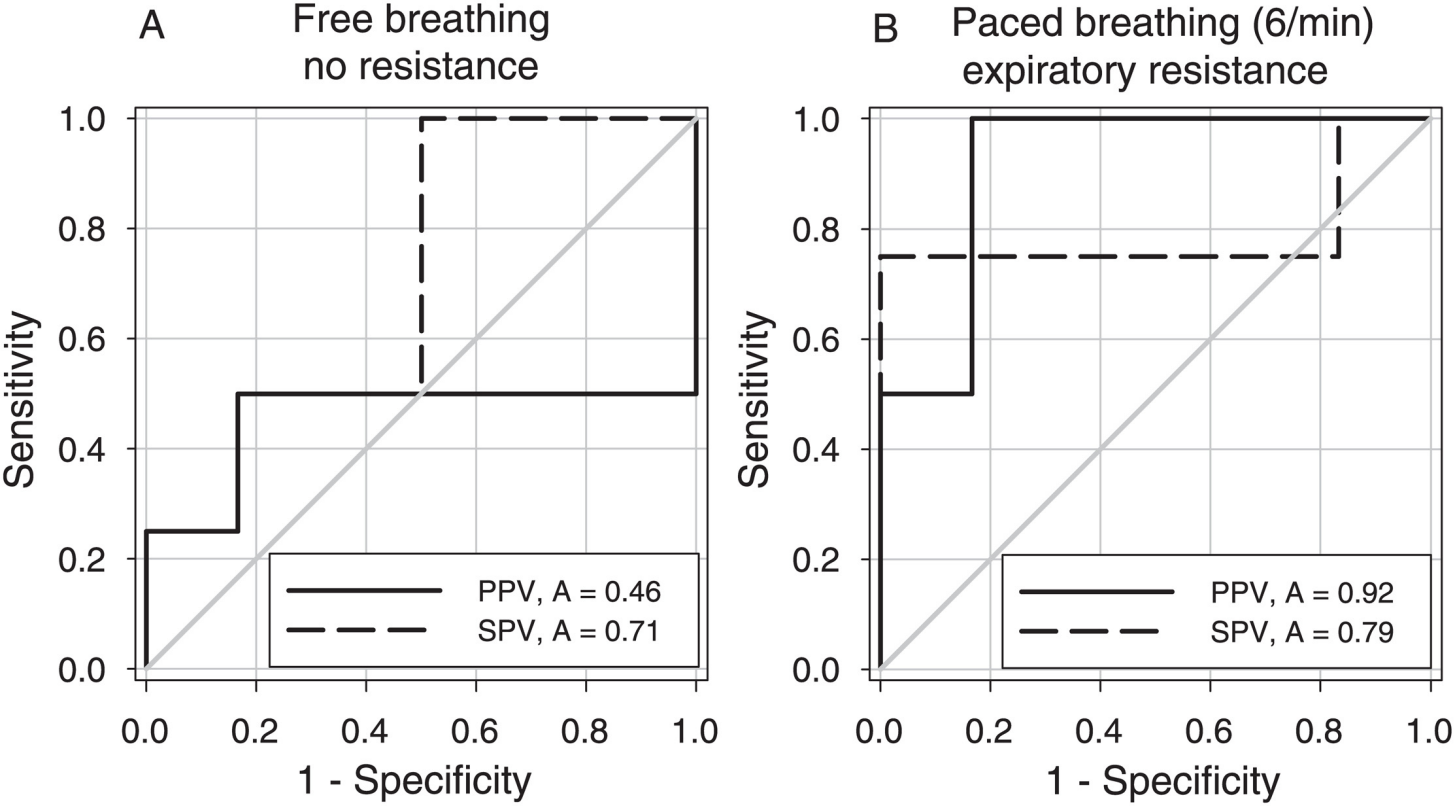
ROC curve plots of pulse (PPV) and systolic pressure variation (SPV) during two different breathing conditions. 6/min paced breathing against an expiratory resistance significantly increased the area under the ROC curve of PPV (p = 0.047).

Compared to free breathing, manipulating breathing conditions by 6/min paced breathing together with an expiratory resistance resulted in higher AUC values for PPV (0.92±0.09 vs. 0.46±0.24, p = 0.047) with no change for SPV (0.79±0.19 vs. 0.71±0.17, p = 0.74), see Fig 4. With specificity set to 83% (5-out-of-6 subjects), sensitivity increased for PPV from 50% (2-out-of-4 subjects) to 100% (4-out-of-4 subjects) and for SPV from 50% (2-out-of-4 subjects) to 75% (3-out-of-4 subjects). Compared to free breathing, 6/min paced breathing with an expiratory resistance increased corresponding cut-off values for PPV (28% vs. 15%) and SPV (17% vs. 7%).

## Discussion

The present study provides insight into arterial pressure variation as biomarker of preload dependency during spontaneous breathing. First, 6/min paced breathing in combination with an external respiratory resistance enhanced the magnitude of PPV and SPV, and thereby improved their discriminative value during progressive central hypovolemia in non-ventilated subjects. Secondly, sensitivity and accuracy of PPV in predicting preload dependency increased.

The current finding that arterial pressure variations, although proven valuable in mechanically ventilated patients, are too small in spontaneously breathing subjects to accurately identify preload dependency [7, 14, 15] conformed to data by Soubrier et al. [14, 15] and Heenen et al. [15] We and others suggest that the respiratory induced changes in intra-thoracic pressure are insufficient to initiate significant preload alterations [14], probably related to breath-to-breath variation in duration and tidal volume [16, 17]. This is compatible with the observation in mechanically ventilated patients, where small tidal volumes and higher respiratory rates masked a preload dependence condition resulting in a lower predictive value of PPV [13, 18, 28–30].

The present study showed less breath-to-breath variation and an increased tidal volume as a result of paced breathing at 6/min. Consequently, slow patterned breathing enhanced both PPV and SPV which is in agreement to earlier findings [31]. Breathing against an external respiratory resistance has been proposed to enhance the respiration induced variation in intra-thoracic pressure and venous return [32, 33]. We hypothesized that the use of an external respiratory resistance could be helpful by initiating a respiratory perturbation that should be great enough to cause significant preload alterations in order to assess its effect on arterial pressure. This would theoretically be more pronounced under circumstances of central hypovolemia. In pigs a respiratory resistor amplified PPV and SPV in response to bleeding [34] and the present study is the first, to our knowledge, to demonstrate this in spontaneously breathing humans.

Our data supports that during spontaneous breathing, enlarging intra-thoracic pressure is a prerequisite to enhance variations in arterial pressure. Paced breathing in combination with artificially increasing changes in airway pressure may be of benefit as a screening tool to assess volume status in perioperative care. Specifically, volume status for the postoperative patient is important, given the high incidence of orthostatic intolerance during early mobilization [35, 36].

### Limitations

Several limitations restrain us from translating the results of this study directly to patient care. First, we studied healthy young adult volunteers rather than patients with compromised cardiovascular function and therefore the results cannot be directly extrapolated to different patient populations. Second, the implementation of slow patterned breathing might be difficult in dyspnoeic patients or patients with altered mental state. Also, the required respiratory threshold resistance may not be optimal for critically ill subjects, although resistance levels up to 10 cmH_2_O are well accepted by most patients treated with continuous positive airway pressure (CPAP) and during breathing physiotherapy in patients pre- and postoperatively [37]. Third, although the fluctuations in both pulse as well as systolic pressure significantly increased by a combination of slow paced breathing together with an external respiratory resistance, ROC analysis confirmed improvement in discriminative value of PPV only. This is probably due to the limited number of subjects included in this study. Nevertheless, the results serve as a proof of principle and we suggest that this approach in non-ventilated subjects merits further study to evaluate its capacity in detecting hypovolemia in a perioperative setting.

In conclusion, our data confirmed the lack of predictive value of arterial pressure variations in spontaneously breathing subjects and demonstrated that paced breathing at 6/min in combination with an external respiratory resistance enhanced the magnitude and discriminative value of PPV during progressive central hypovolemia. Manipulation of breathing conditions in the assessment of hypovolemia in non-ventilated subjects is worthy of further study in a perioperative setting.
